# Resistance training for Black men with depressive symptoms: a pilot randomized controlled trial to assess acceptability, feasibility, and preliminary efficacy

**DOI:** 10.1186/s12888-022-03935-x

**Published:** 2022-04-21

**Authors:** Joseph T. Ciccolo, Mark E. Louie, Nicholas J. SantaBarbara, Christopher T. Webster, James W. Whitworth, Sanaz Nosrat, Michelle Chrastek, Shira I. Dunsiger, Michael P. Carey, Andrew M. Busch

**Affiliations:** 1 Department of Biobehavioral Sciences, Teachers College, Columbia University, 525 West 120th Street, New York, NY 10027 USA; 2 VA Boston Healthcare System, 150 South Huntington Ave, Boston, MA 02130 USA; 3 Behavioral Health Equity Research Group, Hennepin Healthcare Research Institute, 701 Park Ave, Suite PP7.700, Minneapolis, MN 55415 USA; 4 Department of Behavioral and Social Sciences, Brown University School of Public Health, 121 South Main Street, Providence, RI 02903 USA; 5 Department of Psychiatry and Human Behavior, Alpert Medical School Brown University, 700 Butler Dr, Providence, RI 02906 USA; 6 Centers for Behavioral and Preventive Medicine, The Miriam Hospital, 164 Summit Avenue, Providence, RI 02906 USA; 7 Department of Medicine, Hennepin Healthcare, 715 South 8th Street, Minneapolis, MN 55404 USA; 8 Department of Medicine, University of Minnesota Medical School, 401 East River Parkway, Minneapolis, MN 55455 USA

**Keywords:** Depression, Resistance Training, Weightlifting, Behavioral activation

## Abstract

**Background:**

Depression is under-recognized in Black men, who are less likely to seek or have access to psychiatric treatment. Resistance training (RT; i.e., weight lifting) can improve depressive symptoms and may be more acceptable to Black men, but its effects have not been examined for Black men with depressive symptoms.

**Methods:**

Fifty Black men with depressive symptoms were randomized to either (a) 12 weeks of RT (coupled with Behavioral Activation techniques to promote adherence) or (b) an attention-control group (Health, Wellness, and Education; HWE). Both groups met twice/week for 12 weeks, and follow-up assessments were done at end-of-treatment (EOT) and 6 months after enrollment. Changes in physical activity and muscular strength were collected as a manipulation check. The primary outcome was interviewer assessed symptoms of depression using the Quick Inventory of Depression Symptomology (QIDS). Secondary outcomes included self-reported depressive symptoms, anxiety, and stress. The association between change in QIDS from baseline to EOT and concurrent changes in physical activity and muscular strength in the RT group were explored as an initial assessment of mechanism. Longitudinal mixed effects regression models with subject-specific intercepts were used to examine intervention effects.

**Results:**

A sample with high rates of medical comorbidities (e.g., 44% HIV positive), substance use (e.g., 34% smoking), and negative social determinates of health (e.g., 50% unemployed) was enrolled. Recruitment, engagement, and retention data indicate that the intervention and design were feasible. The RT group showed greater gains in self-reported exercise (b = 270.94, SE = 105.69, *p* = .01) and muscular strength (b = 11.71, SE = 4.23, *p* = .01 for upper body and b = 4.24, SE = 2.02, *p* = .04 for lower body) than the HWE group. The RT group had greater reductions in QIDS scores at both EOT (b = -3.00, SE = 1.34, *p* = .01) and 6 months (b = -2.63, SE = 1.81, *p* = .04). The RT group showed a greater reduction in anxiety at EOT (b = -2.67, SE = 1.06, *p* = .02). Findings regarding self-reported depressive symptoms and stress were non-significant, but in the expected direction with effect sizes in the small to medium range. In the RT group, improvement on the QIDS between baseline and EOT was associated with concurrent improvements in physical activity (b = 21.03, SE = 11.16, *p* = .02) and muscular strength (b = 1.27, SE = .44, *p* = .03 for upper body and b = .75, SE = .14, *p* = .03 for lower body).

**Conclusions:**

Results suggest that RT is feasible and may be efficacious for reducing depressive symptoms among underserved urban Black men.

**Trial Registration:**

ClinicalTrial.gov #: NCT03107039 (Registered 11/04/2017).

## Background

Depressive disorders are a leading worldwide cause of disability, affecting approximately 264 million each year [1]. In the United States, about 17.3 million adults (7.1% of the adult population) have at least one major depressive episode in a given year [2]. More women experience depression than men; however, depression is well-known to be under-reported, under-recognized, and seldom treated in men [3–5]. There are numerous reasons for this, but a key factor is that many men view traditional psychiatric treatments (i.e., medication or psychotherapy) as unacceptable [6] or unnecessary [7].

The lack of recognition and treatment for depression is particularly problematic among Black men [Note: The term “Black” is used throughout the paper to include the diverse ways people with origins in any of the Black racial groups of Africa self-identify (e.g., Black, African American, Afro-Caribbean, and African). Many of these groups are well represented in the recruitment area (i.e., New York City); therefore, the recruitment and study materials used the term “Black”.]. Black men are at an increased risk for depressive symptoms [8] and are at the greatest risk for non-detection [9]. When compared to other men, Black men have less access to treatment [10] and may experience symptoms that are more persistent, severe, and disabling [11]. Black men often perceive significant barriers to seeking out depression treatment including stigma, a mistrust of the healthcare system, and pressure to adhere to the social norms around being a strong and/or masculine male [12]. As such, there is a great need to find effective approaches to depression treatment that are both acceptable and accessible to Black men. In this regard, there is a great deal of support for physical exercise as a treatment for depressive symptoms. Most of the research in this area has focused on aerobic exercise (e.g., running, walking); however, resistance training (RT; e.g., weight lifting) also has considerable support.

More specifically, there is now a substantial amount of clinical and observational evidence showing the beneficial effects of RT on depressive symptoms. As with other types of exercise, RT is hypothesized to reduce depressive symptoms via multiple neurobiological and psychosocial changes [13, 14]. The earliest RT studies, often small laboratory trials, indicated that single bouts of RT reduce many of the acute negative affective states frequently reported by those with depression (e.g., depressive symptoms, anxiety) [15]. Epidemiological studies have also reported an inverse relationship between participation in RT and depressive severity and symptoms [16, 17]. A 2018 meta-analysis of 33 randomized controlled trials found RT to be associated with a significant reduction in depressive symptoms [13]. The results showed a moderate effect (delta = 0.66; 95% CI, 0.48–0.83) regardless of the participants’ age, health status, volume of training completed, or objective improvements in strength. Research conducted since continues to support these findings, suggesting that RT participation can be helpful for treating depression [18, 19].

Given these considerations, and other research suggesting that RT may be an exercise modality particularly acceptable to Black men [20], the goal of this study was to investigate the effects of RT on depressive symptoms and explore its potential as a treatment option for community-dwelling Black men with depressive symptoms. The specific details of the study rationale and design for this pilot study have been reported previously [21]. Feasibility data, primary outcome (interviewer rated Quick Inventory of Depressive Symptomatology score), and secondary psychosocial outcomes are reported herein.

## Methods

### Study design

The full study protocol for this study has been previously described [21]. This study tested the effects of RT on interviewer-assessed depressive symptoms using a parallel group, single blind (i.e., assessors blind to condition) design. Participants were randomized (1:1 ratio) into a 12-week RT or an attention-control condition (Health, Wellness, and Education; HWE). Both groups met on-site twice per week during the 12-week program (24 sessions, lasting approximately 60-min). Follow-up assessments were conducted at the end-of-treatment (i.e., 13 weeks post-randomization; EOT) and 6 months (i.e., 6 months post-randomization, 3 months after treatment was completed). Follow-up assessments were completed by blind-to-condition staff. A sample size of 50 was required for 80% power to detect between-group differences in depressive symptoms from baseline to end-of-treatment. Assumptions for this power calculation can be found in the protocol paper [21]. Design and reporting comply with the requirements of the Consolidated Standards of Reporting Trials (CONSORT) guidelines.

### Participants and procedure

Inclusion criteria were: 1) self-reported identification as a cisgender man, 2) ≥ 21 years of age at enrollment, 3) self-reported identification as Black or African-American, and 4) scored ≥ 10 on the Patient Health Questionnaire-9 (PHQ-9) [22] at their initial in person screening visit. Exclusion criteria were: 1) experiencing acute psychosis, mania, or suicidality, 2) currently engaged in any RT or engaging in any other exercise for more than 60 min/week during the prior month, and 3) self-reported medical condition that would make exercise unsafe without a medical screening. Specifically, the American College of Sports Medicine preparticipation screening algorithm [23] was used to exclude men who were characterized as needing medical clearance prior to participating in an exercise program.

Participants were recruited from the community using print (i.e., local newspapers) and online (e.g., craigslist) advertisements, e-mailings using listservs of community partners, and flyers hung or handed out at local venues (e.g., community centers, churches, barbershops) and community events. Interested individuals called the study phone number and were briefly screened by phone to determine potential eligibility. During this screen, participants self-reported their demographics, medical conditions, and current physical activity level, and completed the two item Patient Health Questionaire-2 [24] (PHQ-2), which is routinely used as a pre-screen for depression in medical settings. Those with PHQ-2 scores ≥ 2 were considered to be potentially eligible.

Potentially eligible participants were invited to attend an initial in-person session to complete screening for all inclusion criteria (including completing a screening PHQ-9 to confirm eligibility on depressive symptoms), learn more about the study, and sign an informed consent if they were eligible and wanted to enroll. Those participants who completed the consent process were asked to complete three additional assessment visits prior to being randomized (all baseline data were collected prior to randomization). The Quick Inventory of Depressive Symptomatology interview (QIDS) [25] and the PHQ-9 (i.e., baseline PHQ-9 were administered at the final pre-randomization assessment visit).

The randomization scheme was generated in R, version 3.1.0, on a permutated block randomization procedure with small random sized blocks. Randomization was stratified by screening PHQ-9 score: 10–14 vs. ≥ 15. The randomization sequence was generated by an off-site biostatistician and all others remained blind to this sequence until randomization occurred. Staff opened blinded randomization envelopes at the point of randomization. All treatment visits and outcome assessments were conducted in a university laboratory setting.

### Measures

At baseline, participants’ demographics, medical and psychiatric history, and physical characteristics (e.g., height, body weight) were assessed. Past year drug use was assessed at baseline using the 10-item Drug Abuse Screening Test [26] (DAST-10). The DAST-10 assesses use of a range of illicit substances as well as misuse of prescription medications. Notably, cannabis is included as an illicit substance on the DAST-10. Alcohol and tobacco use are not addressed on the DAST-10. Past year alcohol use was measured with the Alcohol Use Disorders Identification Test [27] (AUDIT). Smoking status was assessed through self-report.

The primary outcome was interviewer-rated depressive symptoms (the “gold standard” for assessing depressive symptoms) as measured by the clinician-rated version of the Quick Inventory of Depressive Symptomatology (QIDS) [25]. Secondary outcomes include the PHQ-9 [22], Generalized Anxiety Disorder scale (GAD-7) [28], and the Perceived Stress Scale (PSS-10) [29]. As a manipulation check, we also report on the change in physical activity and maximal muscular strength between baseline and end-of-treatment. Physical activity was measured in metabolic equivalents (METs; i.e., derived from the frequency, intensity, duration, and type of activity completed) using an interview-administered questionnaire [30]. Physical activity is reported as MET minutes over the last week. Upper (i.e., chest press, measured in pounds) and lower (i.e., leg extension, measured in pounds) body maximal muscular strength were assessed separately by following the gold standard techniques outlined by the National Strength and Conditioning Association [31]. Qualitative interviews were conducted with 7 participants following the 6-month follow-up assessment to gather qualitative experiences with study logistics and treatment conditions. A staff member was trained to conduct these interviews by the last author using a using a semi-structured interview guide. Qualitative interviews were initiated mid-way through the study. Once initiated, qualitative interviews were offered to a random sample of participants following completion of the 6-month follow-up assessment. A summary of the major findings from these interviews are presented below. Adverse events (as defined by the Teacher’s College Institutional Review Board) were monitored throughout the study.

### Study groups

The components of each study arm were detailed previously [21]. A summary of treatment components is provided below. The contact time offered to each treatment group was equal. That is, both groups were offered two 60-min visits per week for 12 weeks (total of 24 sessions offered in both conditions).

#### Resistance training (RT)

The RT intervention has been described previously [21]. It was based on prior research on resistance exercise for depressive symptoms [13], while also adhering to the American College of Sports Medicine’s guidelines for developing and maintaining muscular fitness [32]. The RT condition also incorporated a Behavioral Activation (BA) counseling component into the protocol to facilitate engagement in and maintenance of RT. Participants were asked to attend two onsite, 60-min sessions per week, each session was at least one day apart to allow sufficient recovery. All sessions had two components: (a) 50 min of resistance exercise and (b) 10 min of BA counseling. In addition, up to 50 min of resistance exercise was assigned for at home completion each week.

The 50 min of supervised exercise was a full-body, individualized RT program consisting of 5 min of warm up (i.e., on a stationary bike), 40 min of RT, and 5 min of cool down (i.e., stretching).

Individualization regarding intensity was based on the results of the initial strength test (completed as part of baseline assessments). Individualization regarding movements was based on what equipment and space the participant regularly had access to outside of sessions. This design allowed participants to do the same movements with similar equipment during supervised and at home RT. Additionally, this approach is directly aligned with prior evidence indicating that the change in depressive symptoms in response to RT is not dependent on total training volume or objective improvements in strength [13].

During weeks 1–3, participants were taught the proper form for multiple upper and lower body exercises. During weeks 4–12, participants completed a full-body routine at our facility that was similar to the routine they were asked to complete at home (i.e., one that could be completed in their physical home space). Regardless of the exercises chosen, all participants completed a variety of upper (e.g., bicep curl, lateral raise, bent over row) and lower (e.g., lunge, dumbbell squat, calf raise) body exercises. RT was progressive in that volume and intensity were increased over time according to American College of Sports Medicine guidelines (i.e., 1–3 sets per exercise; muscular fatigue achieved by 10–15 repetitions). To facilitate at home exercise, a variety of resistance bands were provided to participants at session 4 (i.e., week 2) and a set of adjustable dumbbells was provided at session 10 (i.e., week 5) to facilitate completion of at home RT.

The BA component followed established BA for depression goal-setting procedures [33], but focused on completion of at-home RT and recovery from RT (e.g., stretching) as the target behaviors. For patients that struggled with attendance, BA techniques focused on attendance of in-person RT sessions. In the first session, the BA component included a collaborative discussion linking RT to the participant’s personal values. Participant values were revisited throughout the intervention as motivators for treatment engagement and at-home RT. In all RT sessions, the interventionist and participant collaboratively set goals to be completed at home before the next session. Goals were set concretely with agreement on sets, repetitions, logistics (e.g., where and when participant plans to complete), and how to problem-solve expected barriers to completion. Completion of these goals was then reviewed at subsequent in-person sessions. Information from this review often reveled facilitators (e.g., supportive partner) or barriers (e.g., lack of appropriate space in home for assigned movement) in the participant’s environment that could be incorporated into future goal setting.

A workbook was provided to RT participants to record their personal values, weekly RT goals, plans to overcome barriers to weekly RT goals, and to what extent each weekly goal was completed. This workbook helped to guide BA based goal setting in each RT session.

#### Health, wellness, and education

The goal of the HWE intervention was to conduct a time- and attention-matched program with content unlikely to impact primary or secondary study outcomes. HWE consisted of a 30–40 min informational/educational video followed by a 20–30 min discussion of the video (for a total of 60 min of contact). Interactive worksheets and informational handouts were used to facilitate discussion. Participants were invited to take these handouts home following session. The originally planned topics included brain health and functioning, sex and relationships, music history, creativity, and personality types. These topics had been successfully used by the first author in previous studies [34]. As these topics were not originally designed for or tested in Black men with depressive symptoms and the current study was preliminary, we allowed some flexibility in topics based on participant preference. Specifically, if participants reported that some of the original topics did not apply to them, the interventionist would solicit new topics that the participant considered relevant. Then the research team would develop sessions around this topic to offer to the requesting participant as well as future participants. Emergent topics developed through this process included wellness during incarceration, resiliency following incarceration, history of LGBTQ social justice, and history of African American activism. All original and emergent topics were free of any content on depression, exercise, or other topics that could impact secondary outcomes and/or purported treatment mediators [21]. HWE did not include any BA content. HWE participants were not asked to restrict their exercise during the study in any way. To promote equity between conditions, after the final assessment, participants randomized to HWE were provided that same exercise equipment provided to those in the RT group.

### Interventionist background, training, and supervision

The primary interventionist was both a doctoral student in applied exercise physiology and a licensed mental health counselor. This person completed the vast majority (> 95% of sessions). When this primary interventionist was unavailable (e.g., due to illness or time off), sessions were completed by a back-up interventionist who was a doctoral student in applied exercise physiology. Interventionists received didactic and role play training in BA with the last author, a clinical psychologist. Interventionist training in the RT protocol and the HWE topics was conducted by the first author and consisted of didactic, role play, and hands-on training. Interventionists received weekly supervision on BA components from the last author and on RT and HWE components from the first author. Although both interventionists in this study were graduate students, the RT and HWE manuals were designed with the goal that fitness professionals without a college degree (e.g., certified personal trainers, certified strength and conditioning specialists, or similar professionals) would have the background necessary to provide the content with fidelity.

### Intervention fidelity

Interventionists self-rated fidelity to intervention components in both conditions by completing a treatment fidelity checklist. All of the BA content was audio recorded, and 10% of the sessions were reviewed by the last author (a licensed clinical psychologist and BA expert) using the treatment fidelity checklist. In the RT condition interventionists recorded the content and number of at home RT goals set at each session. In subsequent sessions, interventionists recorded the extent to which these goals were completed.

### Incentives

Participants were compensated for completing assessments at baseline ($20), end-of-treatment ($50) and 6-month follow-up ($50). In addition, all participants received $100 worth of home exercise equipment (i.e., free weights, resistance bands). This was given to the RT group during the intervention (i.e., to facilitate home exercise) and to the HWE group after the 6-month follow-up assessment. Finally, to cover public transportation (i.e., bus, subway) costs, all participants were given $5.50 for each on-site randomized session attended. 

### Statistical analysis

Baseline demographics, medical comorbidities, and baseline psychosocial data were summarized by treatment arm and compared using *t*-tests, chi-squared tests and non-parametric tests as appropriate. Correlation analysis was used to identify potential confounders of the treatment effect. Namely, any baseline variable that differed between conditions (at a modest *p* < 0.10 level) and was correlated with the outcome (primary and secondary) at the *r* ≥ 0.10, were considered confounders and adjusted for in the subsequent models.

Using a longitudinal mixed effects regression model with subject-specific intercept, we examined the effect of treatment assigned on the primary outcome (QIDS score at EOT and 6-month follow-up). Models regressed outcomes on treatment, time, treatment*time, baseline value of the outcome and confounders. Models adjusted the standard errors for correlated outcomes within participants over time. Unstandardized effects are reported and can be interpreted on the original scale of the data. Effect sizes (*f*^*2*^*)* have also been included (a value of 0.15 is considered a medium effect).

Next, using a similar analytic approach to that described above (mixed effects regression models), treatment effects on secondary psychosocial outcomes and manipulation check variables were examined. All models included subject specific intercepts, which allow for modeling person-level trajectories over time. Finally, using a series of longitudinal mixed effects model, we examined associations between changes in the QIDS between baseline and EOT and concurrent changes in muscular strength and self-reported exercise, among those randomized to RT. All analyses were conducted in SAS 9.3 and a two-sided significance level was set at 0.05 a priori. Models use a likelihood-based approach to estimation and thus make use of all available data without directly imputing missing outcomes.

## Results

### Sample and baseline characteristics

**Fig. 1:**
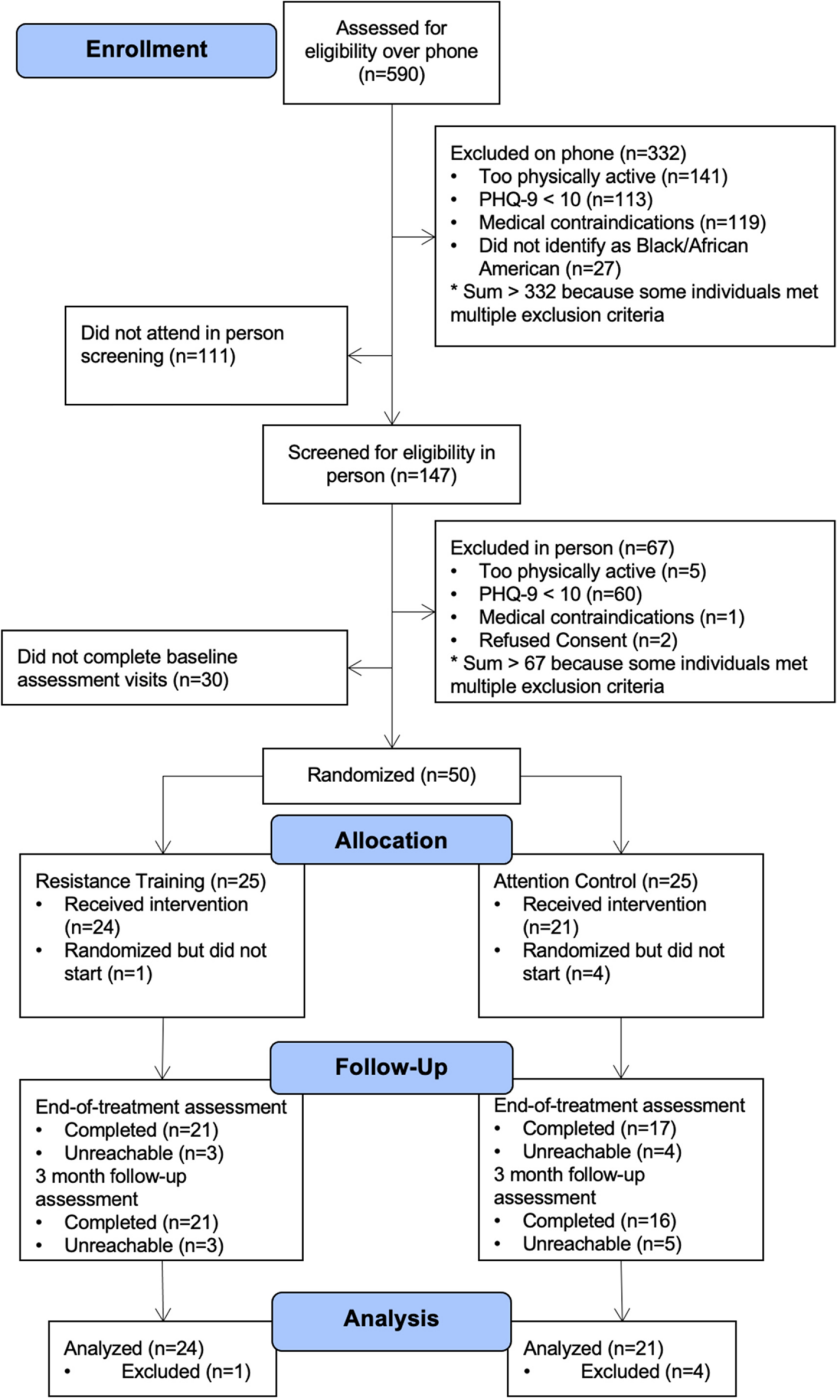
Consort Diagram

A total of 590 participants were screened, 80 completed all screening and qualified for the study, and 50 completed all baseline measurement and were randomized. Twenty-five participants were randomized to each condition. The consort diagram in Fig. 1 provides full details of exclusions.

Participants were 41.34 years of age on average (SD = 12.29), most had not completed a post-secondary degree (64.0% reported not completing a vocational or college degree), 50.0% were unemployed, and 60.0% reporting an annual household income of $10,000 or less. Participants were diverse regarding sexual orientation with 44.0% identifying as straight, 40% identifying as gay, 14% identifying as bi-sexual, and 2% identifying as “other”. 78.0% of the sample identified as Black non-Hispanic/Latino, 14.0% identified as Black Hispanic/Latino, and 8% identified as Black-other (e.g., “Jamaican”). 38.0% of the sample was engaged in psychiatric treatment (i.e., self-reported engagement in counseling and/or psychiatric medication) for a mental illness at baseline. Participants reported a high rate of tobacco, alcohol, and other substance use. 44% of participants reported being HIV positive. A full description by condition is presented in Table 1. There were no significant between-group differences at baseline (*p*s > 0.10), and thus, no statistical confounders included in the models of study outcomes.

**Table 1:** Baseline Characteristics by Study Group, *N* = 50

	RT, *n* = 25	HWE, *n* = 25
Age, years,	41.68(13.44)	41.00(11.29)
Education		
< High School Graduate	1(4%)	4(16%)
High School Graduate	8(32%)	6(24%)
Some Vocational/College	6(24%)	7(28%)
Completed Vocational School	1(4%)	0(0%)
Completed College	5(20%)	3(12%)
Some Graduate School	0(0%)	3(12%)
Completed Graduate School	3(12%)	2(8%)
Refused	1(4%)	0(0%)
Employment		
Full Time	5(20%)	3(12%)
Part Time	2(8%)	5(20%)
Unemployed	14(56%)	11(44%)
Retired	3(12%)	1(4%)
Student	1(4%)	4(16%)
Refused	0(0%)	1(4%)
Annual Household Income ≤ $10,000^a^	12(63%)	12(57%)
Sexual Orientation		
Straight	11(44%)	11(44%)
Gay	8(32%)	12(48%)
Bisexual	6(24%)	1(4%)
Other	0(0%)	1(4%)
Race		
Black/African American, Not Hispanic/Latino	18(72%)	21(84%)
Black/African American, Hispanic/Latino	5(20%)	2(8%)
Black/African American, Other	2(8%)	2(8%)
Medical Co-Morbidities		
HIV/AIDS	10(40%)	12(48%)
Hypertension^b^	3(12%)	2(8%)
Asthma	1(4%)	3(12%)
Pre-Diabetes	1(4%)	1(4%)
Epilepsy	1(4%)	0(0%)
Current Psychiatric Treatment for Depression	7(28%)	7(28%)
Current Psychiatric Treatment for any mental illness (including Depression)	11(44%)	8(32%)
Psychological Co-Morbidity		
Anxiety Disorder	3(12%)	5(20%)
Attention Deficit Hyperactivity Disorder	0	1(4%)
Bipolar Disorder	1(4%)	0
Currently Smoking Cigarettes	7(28%)	10(40%)
Past Year Illicit Drug use on DAST		
No Problems Reported	1(4%)	2(8%)
Low Level	13(52%)	14(56%)
Moderate Level	8(32%)	4(16%)
Substantial Level	2(8%)	4(16%)
Severe Level	1(4%)	1(4%)
Past Year Alcohol use on AUDIT		
Abstainer	7(28%)	8((32%)
Low-Risk Consumption	10(40%)	11(44%)
Hazardous or Harmful Consumption	7(28%)	3(12%)
Moderate-Severe Use Disorder	1(4%)	3(12%)

There were no significant between group differences, *p*s > .05. Means (standard deviations) presented for continuous variables; *n*(%) presented for categorical variables. ^a^*n* = 10 did not respond. ^b^Effectively treated with medications at time of study enrollment

### Feasibility and acceptability

Enrollment occurred between June 2017 and August 2018; we recruited approximately 3 to 4 participants per month. Of the 80 participants who qualified for the study, 50 (62.5%) enrolled. 45 (56.3% of those who qualified) initiated treatment. All results below report on the analyzed sample of 45.

Participants in the RT condition averaged 20.45 sessions completed (SD = 6.15), while those in the HWE condition averaged 15.68 sessions completed (SD = 8.62), *p* = 0.02. A total of 84.4% of participants completed the EOT assessment and 82.2% completed the 6-month assessment.

Post-treatment qualitative interviews were conducted with participants from both the RT (*n* = 4) and the HWE (*n* = 3) groups. All participants expressed satisfaction with study logistical procedures, particularly the flexibility in scheduling (e.g., evening and weekend sessions). The strength testing and other assessments were well-tolerated. All participants reported they were able to understand and provide accurate answers to survey questions. Those in the RT group indicated that training was more physically challenging than anticipated, though they were able to adjust to the program with the help of the interventionist. In addition, there was a clear consensus among RT participants preferring additional training after the 12 weeks offered. Those in the HWE group indicated that the subject matter was generally interesting and that they appreciated the follow-up discussions with program staff. However, they also suggested that HWE engagement and interest could be improved if HWE content was customized to individual interests and lifestyles.

### Manipulation check

Unadjusted exercise time and muscular strength at baseline and EOT are presented in Table 2. As expected, RT participants had greater increases in mean exercise time (MET minutes) from baseline to EOT compared to HWE (b = 270.94, SE = 105.69, *p* = 0.01). Similarly, there were greater increases in muscular strength for RT vs. HWE participants over time (b = 11.71, SE = 4.23, *p* = 0.01 for upper body and b = 4.24, SE = 2.02, *p* = 0.04 for lower body).

**Table 2:** Unadjusted Mean Scores (Manipulation Check, Primary and Secondary Outcomes) Between Groups over Time

	RT	HWE
Exercise Time (MET minutes)^a^		
Baseline	Median = 0 (0–240)	Median = 0(0–200)
EOT	Median = 252.50(0–1845)	Median = 0(0–1120)
Muscular Strength-upper (Pounds)		
Baseline	82.80(29.12)	73.40(28.47)
EOT	94.88(29.21)	71.41(17.32)
Muscular Strength-lower (Pounds)		
Baseline	61.20(18.21)	52.90(15.41)
EOT	62.75(25.73)	50.00(11.11)
QIDS		
Baseline	11.57(3.85)	10.06(2.60)
EOT	5.43(3.09)	6.33(3.81)
6 Months	7.29(5.78)	7.64(4.58)
GAD-7		
Baseline	9.65(5.45)	7.61(5.09)
EOT	4.43(4.27)	4.73(4.92)
6 Months	6.95(5.35)	5.21(6.13)
PHQ-9		
Screening	14.48(4.54)	14.16(4.07)
Baseline	12.18(5.16)	9.67(4.59)
EOT	5.62(5.04)	6.21(5.58)
6 Months	5.57(5.23)	5.14(5.82)
PSS		
Baseline	22.91(5.46)	20.61(5.14)
EOT	17.86(5.67)	17.53(3.40)
6 Months	19.48(8.03)	16.36(5.39)

^a^Unadjusted means (standard deviations) are reported except for Exercise time, which was significantly skewed and thus medians (ranges) are presented

### Primary outcome

**Fig. 2:**
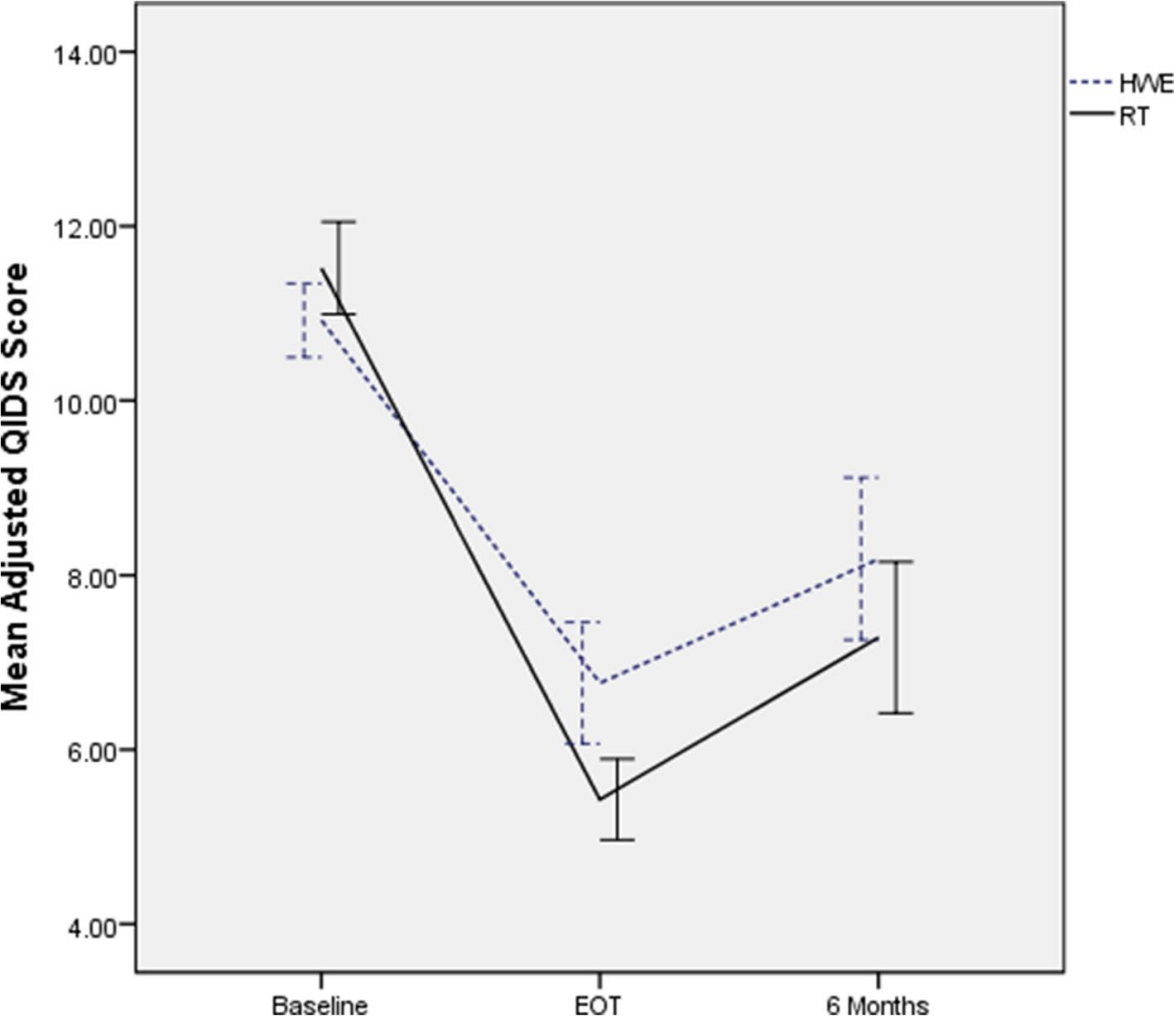
Adjusted QIDS over Time by Group

Unadjusted QIDS scores over time are presented in Table 2. A longitudinal analysis indicated the RT group showed greater reductions in QIDS scores at both EOT (b = -3.00, SE = 1.34, *p* = 0.01, *f*^*2*^ = 0.15) and 6 months (b = -2.63, SE = 1.81, *p* = 0.04, *f*^*2*^ = 0.06), with effect sizes (*f*^2^) in the medium range at EOT and the small range at 6 months. Figure 2 shows adjusted QIDS scores over time by group. Error bars represent 95% confidence intervals for cross-sectional estimates of adjusted mean differences at each follow-up.

### Secondary outcomes

Unadjusted secondary outcomes over time are presented in Table 2. The RT group showed a greater reduction in GAD-7 scores at EOT (b = -2.67, SE = 1.06, *p* = 0.02, *f*^*2*^ = 0.05), but there was no significant difference between groups at 6 months (b = -1.12, SE = 1.81, *p* = 0.54, *f*^*2*^ = 0.01). Results suggest a trend favoring RT with respect to change in PHQ-9 scores at EOT (b = -0.41, SE = 0.47, *p* = 0.08, *f*^*2*^ = 0.14), but no effects at 6 months (b = -0.38, SE = 0.57, *p* = 0.19, *f*^*2*^ = 0.02). Finally, all PSS outcomes were non-significant, but in expected direction (b = -1.97, SE = 2.48, *p* = 0.43, *f*^*2*^ = 0.10 at EOT and b = -0.30, SE = 2.63, *p* = 0.91, *f*^*2*^ = 0.03 at 6 months).

### Changes in QIDS and concurrent changes in muscular strength and self-reported exercise

Among those randomized to the RT group, improvement on the QIDS between baseline and EOT was associated with concurrent improvements in muscular strength (b = 1.27, SE = 0.44, *p* = 0.03, *f*^*2*^ = 0.05 for upper body and b = 0.75, SE = 0.14, *p* = 0.03, *f*^*2*^ = 0.02 for lower body) and self-reported exercise (b = 21.03, SE = 11.16, *p* = 0.02, *f*^*2*^ = 0.14).

### Treatment fidelity and process

On the treatment fidelity checklist, interventionists self-rated that they provided 99.6% of HWE components and 99.8% of planned BA treatment components. Supervisor rated treatment fidelity checklists indicated that the interventionist provided 98.3% of BA treatment components. RT participants set an average of 3.2 (SD = 1.9) at home goals to engage in RT exercises or recovery per in person session attended. RT participants self-reported that they completed 85.9% (SD = 12.0) of the at home goals they set.

### Concurrent treatment

Minimal initiation of outside of study concurrent depression treatment was observed. Specifically, one participant (1 in RT and 0 in HWE) initiated outside of study psychiatric medication for depression between baseline and end-of-treatment. Four participants (2 in RT and 2 in HWE) initiated outside of study counseling for depression between baseline and end-of-treatment.

### Adverse events

No injuries or medical incidents occurred during study activities (i.e., screening assessments, RT sessions, or HWE sessions). No adverse events as defined by the IRB occurred.

## Discussion

In this sample of community-dwelling Black men with mild-to-moderate symptoms of depression, RT resulted in a significantly greater reduction in interview assessed depression symptoms (our primary outcome) at both the end of treatment and 6-month follow-up compared with an attention-control condition. To our knowledge, this is the first study to show such an effect in this population, and the only study to use Behavioral Activation to overcome adherence barriers to RT that are common among those with depressive symptoms. These findings are noteworthy for several reasons.

First, these results suggest that the study design and interventions were feasible and would support a larger trial with this population. More specifically, greater than 60% of the eligible participants enrolled, and session attendance was high, with an about 20 out of 24 sessions being completed by the RT group. Almost 85% of participants who initiated treatment completed the end-of-treatment assessment and approximately 80% completed the 6-month assessment. These rates are similar or better than other RT for depression trials [13]. The RT intervention appears acceptable as indicated by the high rates of session attendance, compliance with at home RT, and results of the qualitative interviews. Further, preliminary mechanism analyses in the RT condition support the hypothesis that increasing physical activity and strength are driving improvements in depressive symptoms. These preliminary mechanism results await replication in formal mediational analyses in a full-scale RCT. Lastly, there were no reported adverse events or effects.

Second, the results suggest that RT is a potentially efficacious treatment option for community-dwelling Black men. The between groups effect size observed in this study (*f*^*2*^ = 0.15) is equivalent to the effect of anti-depressants vs. placebo observed in a recent comprehensive meta-analysis [35]. This is particularly important for this population, because Black men continue to have less access to traditional psychiatric treatments and find such treatments less acceptable. In our sample, all participants were experiencing significant depression symptoms at enrollment, but only 38% were engaged in any psychiatric treatment. RT could offer an alternative for those who may have concerns about traditional psychiatric care or an adjunctive intervention in additional to traditional psychiatric treatments. This could offer Black men an intervention that is less stigmatized as well as more acceptable and accessible.

Third, the results also provide additional empirical evidence to an increasing amount of data showing a robust effect of RT on clinical and subclinical levels of depressive symptoms in adults. The findings of this study are in line with results from a 2018 meta-analysis of 33 RCTs which found a significantly greater reduction in depressive symptoms with RT relative to control [13]. The current findings extend these results to a new population subgroup (Black men) expanding the evidence base of RT for depressive symptoms.

Finally, many men in our sample had significant symptoms of anxiety at baseline and the RT group experienced a significantly greater reduction in anxiety at the end of treatment than HWE. This is notable for several reasons. First, as reported by Bond and colleagues [36], the majority of studies examining the effect of exercise on depressive symptoms have failed to measure or report the percentage of the sample also experiencing anxiety. Second, to our knowledge, this is the only study to examine the effects of RT on anxiety in a sample of Black men. Finally, the effects are similar to other studies testing the impact of RT on anxiety in other populations. For example, prior work has shown RT to reduce anxiety in young [37] and older adults [38], post-stroke patients [39], and those experiencing post-traumatic stress symptoms [40]. Indeed, a 2017 meta-analysis of 16 RCTs [41] reported a medium effect of RT on anxiety among healthy adults and a small effect of RT on anxiety among those with mental or physical illness. The results of the current study fall within that range of effects. Overall, these preliminary findings strongly suggest more work is needed to determine how RT can be useful for reducing anxiety in Black men. 

### Strengths and limitations

Two key strengths of this study were the use of (a) a randomized, attention-controlled design and (b) blinded assessment. In prior research testing the effects of exercise on depressive symptoms, blinded assessment has consistently been shown to be associated with smaller effects. The 2018 meta-analysis on RT for depressive symptoms [13] reported a significant effect of blinding, which is congruent with a 2013 Cochrane Review that identified an attenuation of the effect of exercise on depressive symptoms after excluding trials that did not use blind assessment [42]. Thus, this study adds to the literature supporting a robust effect of RT when assessors were blind to experimental condition. Use of an attention-control increases confidence that the impact on depressive symptoms observed was caused by the RT itself rather than differential attention between conditions. A third important strength is that the intervention manual used in the study was designed to be administered by fitness professionals in the community (i.e., no special counseling or psychiatric training needed). This increases the potential for community dissemination.

In comparison to other research in this area, our sample was characterized by their very low income, high rates of unemployment, and elevated rates of HIV and substance use. Some of these sample characteristics are likely due to the urban setting of New York City and the structural racism that affects Black men (e.g., employment discrimination, lack of access to substance abuse treatment). City level data indicate that that Black New York City residents have significantly lower household incomes and experience a significantly higher unemployment rate than the average New York City Resident [43, 44]. Successful recruitment via flyers at a community/crisis center with a relatively high rate of HIV among their clientele likely contributed to the unintentional over-sampling of men with HIV, but it is also notable that Black men experience the highest prevalence of HIV among residents of New York City [45]. These sample characteristics are a strength of this study in that the needs of an underserved group were addressed, and that we have shown RT to be a promising intervention for depressive symptoms in urban, Black men, even in the context of high rates of co-morbidities and negative social determinates of health. However, the idiosyncratic characteristics of this sample could be viewed as a limitation in that we do not know if our results will generalize to a more representative sample of Black men.

Limitations of this study include 1) that the primary interventionist was a graduate student and licensed mental health professional. Future studies should demonstrate that community fitness professionals can provide the RT manual with fidelity. 2) The small sample and short-term follow-up intervals of this pilot study do not allow for confident conclusions regarding the robustness and longevity of observed effects. Future studies should be fully powered to observe effects on depressive symptoms at longer-term follow-ups (e.g., 1-year post enrollment). 3) Both interventions in this study were provided in a university laboratory setting and significant incentives for participation (e.g., free RT equipment and reimbursement for assessments and transportation) were provided, which limits generalizability to community settings. 4) Finally, attendance was better in the RT condition with approximately 20 sessions attended in the RT condition versus 16 attended in the HWE condition. Thus, while we intended a full attention control, results indicate only a partial attention control was accomplished.

## Conclusions

The results of this study demonstrate the feasibility, acceptability, and preliminary efficacy of using RT as a treatment tool for Black men experiencing depression symptoms. The findings are encouraging and suggest more work is warranted. Future research will be essential for designing community-based RT programs that could eventually increase access to and acceptability of depression care for Black men. Future work could also explore RT as an adjunctive treatment to standard psychiatric care.

## Data Availability

A deidentified version of the dataset used in the current study is available from the corresponding author upon reasonable request.

## Abbreviations

RT: Resistance training
HWE: Health, wellness, and education attention control condition
BA: Behavioral activation
EOT: End-of-Treatment assessment
QIDS: Quick Inventory of Depressive Symptomatology
PHQ-9: Patient Health Questionnaire-9
GAD-7: Generalized Anxiety Disorder scale
PSS-10: Perceived Stress Scale
METs: Metabolic equivalents

## References

[1] World Health Organization. Depression 2020 Available from: https://www.who.int/news-room/fact-sheets/detail/depression.

[2] Villarroel MA, Terlizzi EP (2020). Symptoms of Depression Among Adults: United States, 2019. NCHS Data Brief.

[3] Addis ME, Mahalik JR (2003). Men, masculinity, and the contexts of help seeking. Am Psychol.

[4] González HM, Croghan T, West B, Williams D, Nesse R, Tarraf W (2008). Antidepressant use in black and white populations in the United States. Psychiatr Serv.

[5] González HM, Tarraf W, Whitfield KE, Vega WA (2010). The epidemiology of major depression and ethnicity in the United States. J Psychiatr Res.

[6] Rice SM, Oliffe JL, Kealy D, Seidler ZE, Ogrodniczuk JS (2020). Men's help-seeking for depression: Attitudinal and structural barriers in symptomatic men. J Prim Care Community Health.

[7] Krumm S, Checchia C, Koesters M, Kilian R, Becker T (2017). Men's views on depression: A systematic review and metasynthesis of qualitative research. Psychopathology.

[8] Thorpe RJ, Whitfield KE (2018). Psychosocial influences of African Americans men's health. J Gerontol B Psychol Sci Soc Sci.

[9] Gayman MD, Lennox Kail B, Spring A, Greenidge GR (2018). Risk and protective factors for depressive symptoms among African American men: An application of the Stress Process Model. J Gerontol B Psychol Sci Soc Sci.

[10] Blumberg SJ, Clarke TC, Blackwell DL (2015). Racial and ethnic disparities in men's use of mental health treatments. NCHS Data Brief.

[11] Williams DR, González HM, Neighbors H, Nesse R, Abelson JM, Sweetman J (2007). Prevalence and distribution of major depressive disorder in African Americans, Caribbean blacks, and non-Hispanic whites: results from the National Survey of American Life. Arch Gen Psychiatry.

[12] Hudson DL, Eaton J, Banks A, Sewell W, Neighbors H (2018). "Down in the Sewers": Perceptions of depression and depression care among African American men. Am J Mens Health.

[13] Gordon BR, McDowell CP, Hallgren M, Meyer JD, Lyons M, Herring MP (2018). Association of efficacy of resistance exercise training with depressive symptoms: Meta-analysis and meta-regression analysis of randomized clinical trials. JAMA Psychiat.

[14] De Sousa RAL, Rocha-Dias I, de Oliveira LRS, Improta-Caria AC, Monteiro-Junior RS, Cassilhas RC (2021). Molecular mechanisms of physical exercise on depression in the elderly: a systematic review. Mol Biol Rep.

[15] Doyne EJ, Ossip-Klein DJ, Bowman ED, Osborn KM, McDougall-Wilson IB, Neimeyer RA (1987). Running versus weight lifting in the treatment of depression. J Consult Clin Psychol.

[16] Bennie JA, Teychenne MJ, De Cocker K, Biddle SJH (2019). Associations between aerobic and muscle-strengthening exercise with depressive symptom severity among 17,839 U.S. adults. Prev Med.

[17] Bennie JA, De Cocker K, Biddle SJH, Teychenne MJ (2020). Joint and dose-dependent associations between aerobic and muscle-strengthening activity with depression: A cross-sectional study of 1.48 million adults between 2011 and 2017. Depression and anxiety.

[18] Nosrat S, Whitworth JW, SantaBarbara NJ, Dunsiger SI, Ciccolo JT. Acute effects of resistance-exercise intensity in depressed Black/African Americans living with HIV: A randomized pilot study. J Sport Exerc Psychol. 2019;41(5):261–70. 10.1123/jsep.2018-0301 31387082

[19] Moraes HS, Silveira HS, Oliveira NA, Matta Mello Portugal E, Araújo NB, Vasques PE (2020). Is strength training as effective as aerobic training for depression in older adults? A randomized controlled trial. Neuropsychobiology.

[20] Griffith DM, Bergner EM, Cornish EK, McQueen CM (2018). Physical Activity Interventions With African American or Latino Men: A Systematic Review. Am J Mens Health.

[21] Busch AM, Louie ME, SantaBarbara NJ, Ajayi AA, Gleason N, Dunsiger SI (2019). Effects of resistance training on depression and cardiovascular disease risk in Black men: Protocol for a randomized controlled trial. Ment Health Phys Act.

[22] Kroenke K, Spitzer RL, Williams JB (2001). The PHQ-9: validity of a brief depression severity measure. J Gen Intern Med.

[23] Riebe D, Ehrman JK, Liguori G, Magal M, Medicine ACoS. ACSM's guidelines for exercise testing and prescription. Philadelphia: Wolters Kluwer; 2018.

[24] Gilbody S, Richards D, Brealey S, Hewitt C (2007). Screening for depression in medical settings with the Patient Health Questionnaire (PHQ): a diagnostic meta-analysis. J Gen Intern Med.

[25] Rush AJ, Trivedi MH, Ibrahim HM, Carmody TJ, Arnow B, Klein DN (2003). The 16-Item Quick Inventory of Depressive Symptomatology (QIDS), clinician rating (QIDS-C), and self-report (QIDS-SR): a psychometric evaluation in patients with chronic major depression. Biol Psychiatry.

[26] Skinner HA (1982). The drug abuse screening test. Addict Behav.

[27] World Health O (2001). AUDIT: the Alcohol Use Disorders Identification Test : guidelines for use in primary health care / Thomas F. Babor.

[28] Spitzer RL, Kroenke K, Williams JB, Löwe B (2006). A brief measure for assessing generalized anxiety disorder: the GAD-7. Arch Intern Med.

[29] Cohen S (1988). Perceived stress in a probability sample of the United States. The social psychology of health. The Claremont Symposium on Applied Social Psychology..

[30] Pettee Gabriel K, McClain JJ, Schmid KK, Storti KL, Ainsworth BE (2011). Reliability and convergent validity of the past-week Modifiable Activity Questionnaire. Public Health Nutr.

[31] Haff GG, Triplett NT. Essentials of strength training and conditioning 4th edition. Champaign: Human kinetics; 2015

[32] American College of Sports Medicine position stand (2009). Progression models in resistance training for healthy adults. Med Sci Sports Exerc.

[33] Kanter JW, Busch AM, Rusch LC. Behavioral activation: Distinctive features. London: Routledge Press; 2009.

[34] Ciccolo JT, Dunsiger SI, Williams DM, Bartholomew JB, Jennings EG, Ussher MH (2011). Resistance training as an aid to standard smoking cessation treatment: A pilot study. Nicotine Tob Res.

[35] Cipriani A, Furukawa TA, Salanti G, Chaimani A, Atkinson LZ, Ogawa Y (2018). Comparative efficacy and acceptability of 21 antidepressant drugs for the acute treatment of adults with major depressive disorder: a systematic review and network meta-analysis. Lancet (London, England).

[36] Bond G, Stanton R, Wintour SA, Rosenbaum S, Rebar AL (2020). Do exercise trials for adults with depression account for comorbid anxiety? A systematic review. Ment Health Phys Act.

[37] Gordon BR, McDowell CP, Lyons M, Herring MP (2020). Resistance exercise training for anxiety and worry symptoms among young adults: a randomized controlled trial. Sci Rep.

[38] Tsutsumi T, Don BM, Zaichkowsky LD, Takenaka K, Oka K, Ohno T (1998). Comparison of high and moderate intensity of strength training on mood and anxiety in older adults. Percept Mot Skills.

[39] Aidar FJ, de Oliveira RJ, Silva AJ, de Matos DG, MaziniFilho ML, Hickner RC (2012). The influence of resistance exercise training on the levels of anxiety in ischemic stroke. Stroke Res Treat.

[40] Whitworth JW, Nosrat S, SantaBarbara NJ, Ciccolo JT. Feasibility of resistance exercise for posttraumatic stress and anxiety symptoms: A randomized controlled pilot study. J Trauma Stress. 2019;32(6):977–84. 10.1002/jts.22464 31743507

[41] Gordon BR, McDowell CP, Lyons M, Herring MP (2017). The effects of resistance exercise training on anxiety: A meta-analysis and meta-regression analysis of randomized controlled trials. Sports Med (Auckland, NZ).

[42] Cooney G, Dwan K, Greig C, Lawlor D, Rimer J, Waugh F (2013). Exercise for depression. Cochrane Database Syst Rev.

[43] New York State Department of Health. New York City Health Indicators by Race/Ethnicity, 2016–2018. Accessed 3/10/22 from www.health.ny.gov/statistics/community/minority/county/newyorkcity.htm

[44] Parrott, JA. New York State’s Unprecedented Covid-19 Unemployment Crisis Requires a Comprehensive, Immediate Active Labor Market Response. A policy brief by The New School Center for New York City Affairs. 2022. Accessed 3/10/22 from www.centernyc.org/reports-briefs/new-york-states-unprecedented-covid-19-unemployment-crisis-requires-a-comprehensive-immediate-active-labor-market-response

[45] Wiewel EW, Hanna DB, Begier EM (2011). High HIV Prevalence and Diagnosis Rates in New York City Black Men. J Community Health.

